# Effect of the Incorporation of Polycaprolactone (PCL) on the Retrogradation of Binary Blends with Cassava Thermoplastic Starch (TPS)

**DOI:** 10.3390/polym13010038

**Published:** 2020-12-24

**Authors:** José Herminsul Mina Hernandez

**Affiliations:** Group Materiales Compuestos, Universidad del Valle, Calle 13 No. 100-00, Cali 76001, Colombia; jose.mina@correounivalle.edu.co; Tel.: +57-2-3212170; Fax: +57-2-3392450

**Keywords:** thermoplastic starch, cassava starch, retrogradation, polycaprolactone, polymer characterization

## Abstract

The effects of incorporating polycaprolactone (PCL) in three binary blends with cassava thermoplastic starch (TPS) at TPS/PCL ratios of 60/40, 50/50, and 40/60 were studied. TPS previously obtained by single-screw extrusion was manually mixed with PCL and then transformed by extrusion. The results’ analysis focused mainly on monitoring the retrogradation phenomenon in TPS for different storage times at two relative humidities (29% and 54%) and constant temperature (25 °C). With the plasticization of the starch, a predominantly amorphous mass was generated, as evidenced by the scanning electron microscopy (SEM), X-ray diffraction (XRD), and Fourier transform infrared (FTIR) results. The results suggested that two opposite processes coexisted simultaneously: retrogradation, which stiffened the material, and plasticization, which softened it, with the latter mechanism predominating at short times and reversing at longer times. With the incorporation of PCL, immiscible blends were obtained in which TPS was the dispersed phase; the mechanical properties improved with the amount of PCL added. The properties of the binary blends as a function of time showed a trend similar to that observed for TPS alone; this finding indicated that the TPS/PCL interactions were not strong enough to affect the structural changes in the TPS, which continued to occur regardless of the PCL content. Finally, it was found that for the binary blend, the relative humidity during storage was more significant to the retrogradation phenomenon than the amount of PCL.

## 1. Introduction

Thermoplastic starch (TPS) is an environmentally friendly alternative in applications of high-turnover products that are currently mostly made with conventional synthetic plastics [1,2,3,4,5]. In addition to the decrease in pollution with the use of this type of polymer, the renewable nature of the raw materials of TPS, which does not depend on oil, is advantageous [6]. Among the starches used, the one based on Cassava (*Manihot esculenta Crantz*) stands out for used as a staple food for human consumption in tropical and subtropical regions, as well as in numerous industrial applications like TPS [7]. Despite the aforementioned advantages, the mass application of TPS is limited by its poor mechanical properties and high moisture adsorption, coupled with the structural instability resulting from changes in these properties as a function of time and the relative humidity to which they are exposed. These changes are based on the recrystallization tendency of amylose and amylopectin experienced by TPS, following the predominantly amorphous state previously achieved with the plasticization process [8,9,10,11]. When heated starch granules are in excess water, they undergo an order–disorder phase transition called gelatinization. This transition occurs over a temperature range that is characteristic of the botanical source of starch. Depending on some processing and storage conditions, such as temperature and humidity, amorphous starch undergoes structural changes after cooling, including recrystallization of amylose and amylopectin into different crystalline structures, phase separation, and polymer reorientation. The molecular interactions (mainly hydrogen bonds between starch chains) that occur after cooling are called retrogradation [12]. This retrogradation also refers to the gelatinized starch changes from an initial amorphous state to a more ordered crystalline form. This process occurs because starch gels are not thermodynamically stable. According to Gudmundsson [13], amylopectin chains are responsible for the retrogradation phenomena generated in the long term, while amylose is related to changes in shorter times. According to the above, one of the first strategies of researchers seeking to increase the stability of secondary interactions with starch consisted of the study of new plasticizers, mostly nitrogenous ones, instead of glycerol, which causes TPS recrystallization over time [1,3,14,15]. Note that although these nitrogenous additives seem to lead to a more stable material in principle, there is currently little knowledge about their influence on the biodegradation of TPS. Another strategy that has been used is the incorporation of fibers and nanofibers to simultaneously attack the problems of retrogradation and the poor mechanical properties of the material [16,17]. This methodology is dependent on the interfacial nature of TPS-reinforcement and on the technological possibilities of manufacturing composites reinforced with discontinuous fibers, where it is difficult to achieve high incorporations of reinforcements at significant aspect ratios. Finally, blends of TPS with equally biodegradable but more stable polymers have been formed [18]. Prominent among these is polycaprolactone (PCL), due both to its biodegradation capacity and its good compatibility with a vast number of polymers, PCL is a synthetic aliphatic polyester that is synthesized from a ring-opening polymerization (ROP) with ε-caprolactone as the base monomer by heating or using catalysts [19,20]. PCL is a partially crystalline polymer with a moderate melting temperature of 60 °C. The material is susceptible to the growth of fungi and bacteria. PCL biodegradation has widely studied, finding that it is degraded by the attack of enzymes, such as lipases, causing damage predominantly in the amorphous regions of the material [21]. In comparison with other aliphatic polyesters, the superior rheological and viscoelastic behavior render PCL easy to process in the manufacture of biodegradable devices, standing out on drug delivery, tissue engineering, and packing applications. Due to its mechanical properties and affinity with starch, has been selected in studies typically focused on the physical, thermal, and mechanical characterization or biodegradation of the blend [22,23,24,25,26,27,28]. However, evaluations at different times are not included to show whether the phenomenon of retrogradation in the TPS/PCL binary blends is maintained. Due to the importance of expanding the knowledge related to the variations in the TPS properties over time and quantifying the possible performance improvement when TPS mixed with a more stable biodegradable polymer, this study presents the results obtained from the preparation and physicochemical and mechanical characterization of a cassava TPS, and the effect of the incorporation of PCL in binary TPS/PCL blends at ratios of 60/40, 50/50, and 40/60 concerning the total mass. The study mainly aimed to understand the material’s structural changes as a function of time by monitoring the retrogradation during storage at two relative humidity values (54 and 29%), keeping the temperature constant (25 °C). 

## 2. Materials

The cassava (*Manihot esculenta Crantz*) starch used to obtain TPS was a carbohydrate free of toxic or harmful substances obtained by wet grinding of cassava for food application. The cassava was a CM 4919 variety with an amylose content of 22–26% and amylopectin content of 78–74%. This material was supplied by the company Ingredion Colombia SA of Cali, Colombia, and was in semicrystalline particles with spherical geometry and an average diameter of 14.44 µm. The glycerol used as plasticizer was three-functional alcohol is a colorless liquid of medium viscosity. This industrial grade material had a purity of 98% and was acquired from Farmacias Comercio, Méridacity, Mexico. The PCL used to prepare the binary TPS/PCL blend was CAPA 6800 from the company Perstorp UK Limited of Warrington, England. This polyester had a molecular mass of 80,000 g/mol, a glass transition temperature (Tg) of −45 °C, a melting temperature (Tm) of 56 °C, and a maximum mass loss rate at 453 °C and was acquired as opaque white pellets. 

## 3. Experimental Procedure

### 3.1. Obtaining the TPS

Similar to the process reported by Huang et al. [1] and Ma et al. [15], the cassava starch, after 24 h of drying at 80 °C, was premixed with glycerol at a 70:30 ratio by mass. This process was carried out for 5 min using a Black and Decker high-speed mixer, until the material had no lumps. Subsequently, the blend was stored in closed polypropylene containers for 72 h. Finally, the material was plasticized in a single-screw extruder coupled to a Brabender PLE-330 plasticorder (Duisburg, Germany), with a 19-mm diameter screw, 4:1 compression ratio, and 25 L/D ratio. The turning speed was maintained at 45 rpm, and the temperature profile was 115, 125, 130, and 135 °C for the three screw zones and head, respectively. The TPS obtained was pelletized and then ground until it passed through a sieve with an average opening of 1 mm. The test specimens were formed in a Carver MH 4389-4021 semiautomatic press (Wabash, IN, USA) equipped with heating plates and a forced water circulation cooling system. To shape the samples, stainless steel molds were used to generate square TPS plates with widths and thicknesses of 120 and 1 mm, respectively. The molding was performed at a temperature of 160 °C, clamping force of 7000 pounds, and a duty cycle of 50 min, which included 30 min of heating and 20 min of cooling under pressure. Finally, the plates were demolded and adjusted to the standardized dimensions, according to the standard for tensile tests ASTM D638-14 [29], with the help of a pneumatic press equipped with a standardized die. The TPS samples obtained were dried at 60 °C for 12 h and subsequently stored at two different relative humidities in glass desiccators containing magnesium nitrate hexahydrate (54 ± 2%) and potassium acetate (29 ± 2%) salts, as specified in ASTM E104-02 [30], keeping the samples at a constant temperature of 25 ± 2 °C. The test specimens were retrieved after different storage times for physical–chemical and mechanical tests.

### 3.2. Preparation of the Binary Blends TPS/PCL

After drying at 50 °C in a vacuum oven, the previously pelletized TPS was manually mixed with the PCL granules at TPS/PCL ratios of 40/60, 50/50, and 60/40 (by mass). These values were selected based on similar studies published in the literature [23,24,25]. Subsequently, this material was extruded in the same equipment as that previously described for obtaining TPS from cassava starch. In this case, the processing conditions were a turning speed of 45 rpm and a temperature profile of 115, 125, 130, and 130 °C for the three screw zones and head, respectively. At the end of the process, the TPS/PCL blend was pelletized and prepared for use in a subsequent hot compression molding at 160 °C with a total cycle of 50 min, consisting of 30 min of heating and 20 min of cooling, at a load of 7000 pounds, with which 120-mm square plates were formed, with a thickness of 1 mm. These plates were punched to obtain test specimens based on the ASTM D638-14 standard [29] for performing tensile tests. As with the TPS samples, the TPS/PCL binary blends were stored at 29% and 54% relative humidity, and physical–chemical and mechanical tests were performed at different storage times.

### 3.3. FTIR Spectroscopy 

A Nicolet Protege 460 Magna-IR spectrometer (Waltham, MA, USA) was used for Fourier transform infrared (FTIR) spectroscopy. For the analysis of the unplasticized starch, the KBr tablet technique was used, whereas for the TPS samples and the binary blend with PCL, an Inspect IR Plus microscope was used, equipped with an attenuated total reflectance (ATR) accessory. The analysis was performed with 100 scans and a resolution of 4 cm^−1^. 

### 3.4. Moisture Adsorption

The TPS and TPS/PCL binary blend samples were dried in an oven at 60 °C for 24 h and then stored at 54 ± 2% and 29 ± 2% relative humidity using desiccators containing magnesium nitrate hexahydrate and potassium acetate salts, respectively, following the ASTM standard E104-02 [30]. Data on the mass gain as a function of time (*M_t_*) were taken at a temperature of 25 ± 2 °C. The moisture adsorption (*H*) was calculated as a percentage, taking as the mass obtained after oven drying (*M_d_*) as the initial weight, as specified in Equation (1).

(1) $$H=\left(\frac{Mt-Md}{Md}\right)\times 100$$

Last, after the moisture adsorption versus time curves were constructed, the adsorption isotherms corresponding to the TPS and the binary blends were obtained.

### 3.5. Scanning Electron Microscopy (SEM)

The morphology of unplasticized cassava starch particles and the fracture surface of the TPS and TPS/PCL binary blends were observed through scanning electron microscopy (SEM) with a JEOL LV 5400 scanning electron microscope (JEOL, Mexico City, Mexico) operated at 20 keV. The samples were coated with gold prior to analysis. Before the analysis, the test specimens were stored in a desiccator at a relative humidity of 54% and a temperature of 25 °C and were evaluated when the moisture adsorption reached its equilibrium value.

### 3.6. Thermogravimetric Analysis (TGA)

The thermal stability of thermoplastic starch, polycaprolactone, and TPS/PCL binary blends was studied using a Perkin Elmer TGA 7 thermogravimetric analysis instrument (PerkinElmer, Inc., Waltham, MA, USA) in a temperature range of 50 to 650 °C, with a heating rate of 10 °C/min and in an inert nitrogen atmosphere with a flow rate of 20 mL/min. The average weight of the samples tested was 7 mg.

### 3.7. X-ray Diffraction (XRD)

The X-ray diffraction (XRD) spectra of the unplasticized cassava starch, TPS and binary blends of the TPS and PCL were obtained in a Siemens D-5000 diffractometer with Bragg–Brentano geometry using CuKα radiation (λ = 1.5418 Å) generated at 34 kV and 25 mA. The test specimens were placed in a rotating sample holder (30 RPM) and recorded in an angular interval of 2° ≤ 2θ ≥ 60°, with a step size of 0.02° and a step time of 3 s. The relative crystalline content of the samples was estimated following the method established by Nara and Komiya [31]. For this purpose, baselines were drawn in the X-ray diffractograms at an interval between 4° and 30° (2θ). Subsequently, curved lines were formed, taking as reference the bases of the peaks of the diffractograms. The area located between the curved line and the baseline was defined as the amorphous area (*A_A_*); likewise, the portion between the curved line and the space formed by the peaks corresponded to the area of the crystalline region (*A_C_*). These areas were determined using Origin^®^ software version 8.0 (OriginLab Corporation, Northampton, MA, USA), and the percentage of relative crystallinity (*C_%_*) was calculated according to the model established in Equation (2).

(2) $$C_{\%}=\left(\frac{A_{C}}{A_{C}+A_{A}}\right)$$

### 3.8. Tension Test

The tensile mechanical properties of the TPS and TPS/PCL binary blend samples were determined. The mechanical evaluation was carried out at different storage times, two relative humidities of 54% and 29% and constant temperature of 25 °C. The tests were carried out in a Shimadzu AG-1, 100 kN universal mechanical testing machine (Nakagyo-ku, Kyoto, Japan), equipped with a 500-N load cell. Type IV specimens were used and tested with a crosshead displacement speed clamps of 5 mm/min, following the ASTM D-638 standard [29]. 

## 4. Results and Discussion

### 4.1. FTIR Spectroscopy

**Figure 1:**
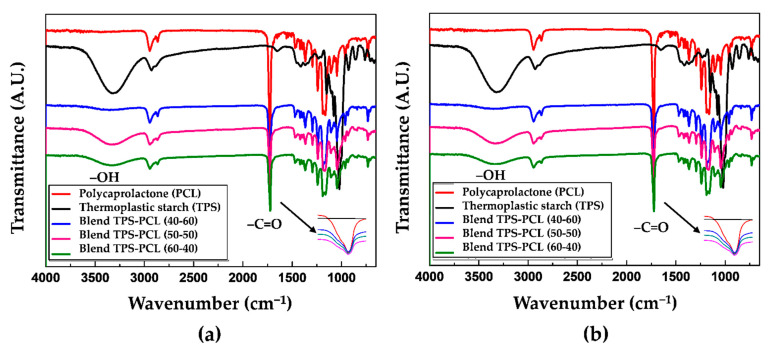
Fourier transform infrared spectroscopy (FTIR) for the polycaprolactone (PCL), thermoplastic starch (TPS), TPS/PCL 40/60 binary blend, TPS/PCL 50/50 binary blend, and TPS/PCL 60/40 binary blend, conditioned at: (**a**) 54% and (**b**) 29%.

Figure 1a shows the FTIR spectra for the TPS and TPS/PCL binary blends at the 40/60, 50/50, and 60/40 ratios (by mass). This material was stored for 3 days at a relative humidity of 54%. The spectra show a gradual increase in the band at 3330 cm^−1^ corresponding to the hydroxyl (–OH) groups as the TPS content in the material increases. Table 1 shows the assignment of the highest intensity bands found in the FTIR spectrum of the TPS/PCL 50/50 blend corresponding to the stretching of the O–H hydroxyls of starch at 3331 cm^−1^, the stretching of the C–H bond of PCL at 2945 cm^−1^, the stretching of the C=O carbonyl of the ester group of PCL at 1724 cm^−1^, the stretching of the C–O–C bond of PCL at 1242 cm^−1^, and the stretching of the C–O–C glycosidic bond of starch at 1043 cm^−1^. The signals strongly coincide with the results reported by Balmayor et al. [32] for a corn starch/PCL (30/70) blend characterized by FTIR using the KBr pellet technique. As a result that TPS has hydroxyl groups, which can be considered proton donors, and PCL has carbonyl groups classified as proton acceptors, in principle, the binary blend of these materials could present hydrogen bonds involving the possible formation of a blend with some degree of miscibility. However, Figure 1a shows that the band corresponding to the stretching vibration of the C=O carbonyls does not shift in any of the three spectra of the TPS/PCL binary blends, identified in the (c), (d), and (e) curves, relative to pure PCL. This lack of shifting indicates that probably few secondary interactions based on hydrogen bonds are generated, and therefore, the system is characterized as being predominantly immiscible. Note that for similar binary blend systems, Cesteros [33] proposed a relationship between the miscibility of polymer blends and shifts in the carbonyl band at lower wavenumbers. In the case of the material stored at 29% relative humidity (Figure 1b), the same bands are observed as in the samples that were stored at 54%. The only difference is the decrease in the height of the band corresponding to the hydroxyl groups in the binary blends, which is due to the lower amount of water adsorbed by TPS at this relative humidity. Similarly, in the specimens stored at 54%, there are no shifts in the carbonyl bands associated with the binary blend, so there is no significant evidence of the establishment of hydrogen bonds between the phases that make up the material. 

### 4.2. Scanning Electron Microscopy (SEM)

**Figure 2:**
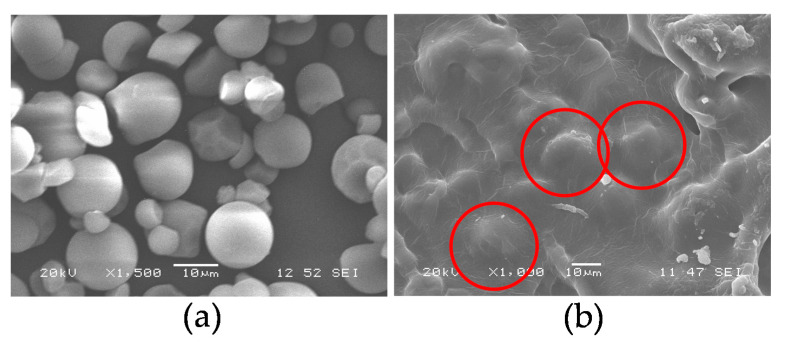
Scanning electron images of: (**a**) unplasticized starch particles; (**b**) fracture surfaces at tension of the TPS at 54% relative humidity and 17 days of storage. The red circles show a few granules that are not completely destructured.

Figure 2a shows a micrograph of the morphology corresponding to the cassava starch particles used in the plasticization process. It was found that some of the particles presented spherical and superficially smooth forms, while in others, truncated forms were evident in both cases, with an average diameter of 14.4 µm. These results were similar to those reported by Cuenca et al. [34] who found particles with similar morphologies and diameters that varied between 5 and 15 μm in starch studies from cassava as a botanical source. Likewise, Rodrigues Dos Santos et al. [35] found an average particle size of 15.7 μm and shapes similar to those of the present work. On the other hand, according to Wurzburg [36], the geometry of this type of starch tends to be spherical, different from other botanical sources, which can exhibit elliptical, oval, lenticular, or polygonal shapes. The presence of the plasticizer and the shear stresses typical of the extrusion plasticization process led to the rupture of the granular structure of the starch. However, according to the micrograph in Figure 2b, there is evidence of a few granules that are not completely destructured shown with the red circle, indicating that it is possible to improve the process conditions followed in the production of the material. This last micrograph is a cross-section of a TPS specimen, previously tested under stress, after 17 days of storage at 54% relative humidity.

**Figure 3:**
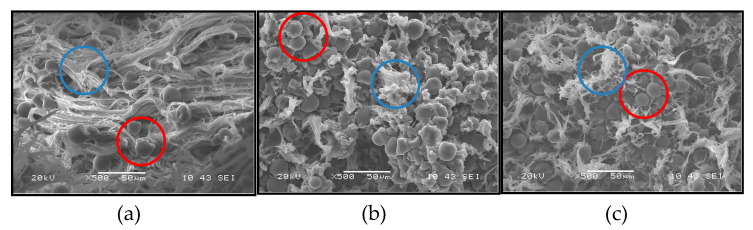
Scanning electron images of the fracture surface of: (**a**) TPS/PCL 40/60 binary blend; (**b**) TPS/PCL 50/50 binary blend; and (**c**) TPS/PCL 60/40 binary blend. The red circles highlight the dispersed phase constituted by the TPS and the red blue highlighted the continuous PCL.

When the TPS is processed by extrusion together with the PCL, an immiscible blend is achieved as shown in the images of the images presented in Figure 3, correspond to the tensile fracture surface of the test specimens manufactured with the TPS/PCL binary blend at the three ratios employed. Here, for all the compositions, the formation of a continuous PCL phase (highlighted with blue circles) and a dispersed TPS phase (highlighted with red circles), randomly distributed, are observed, clearly showing the predominantly immiscible nature of the material. Note that the PCL is the matrix for all binary blends, even for the TPS/PCL 60/40 ratio, where PCL is present in a lower proportion than TPS. This behavior occurs because of the low melting temperature (56 °C) of PCL; under the processing conditions, PCL had greater fluidity than TPS and tended to surround the TPS particles during mixing, constituting the continuous phase. 

The effect of viscosity and cutting speed on the morphology of binary blends has been studied by Tsuji et al. [37], who indicated that the component at the highest proportion is not necessarily the continuous phase. Similarly, Correa et al. [3] evaluated blends of PCL with TPS of corn plasticized with urea and found that the resulting material was completely immiscible and was based on a PCL matrix with TPS as the dispersed phase. For sago starch-PCL blends, at ratios similar to those used in the present study, Ishiaku et al. [38] observed the formation of two phases from the binary blend based on SEM images. The researchers indicated that in the rupture surface of a sample previously tested under stress, there were holes or grooves that were evidence of detachments or tears in the dispersed phase composed of starch, and the morphologies obtained were similar to those shown in Figure 3. It was proposed that with the prolonged heating of the starch and the PCL during molding, water vapor was formed, which in turn generated voids around the starch particles, reducing the interfacial adhesion and negatively affecting the mechanical properties of the material.

### 4.3. Moisture Adsorption

**Figure 4:**
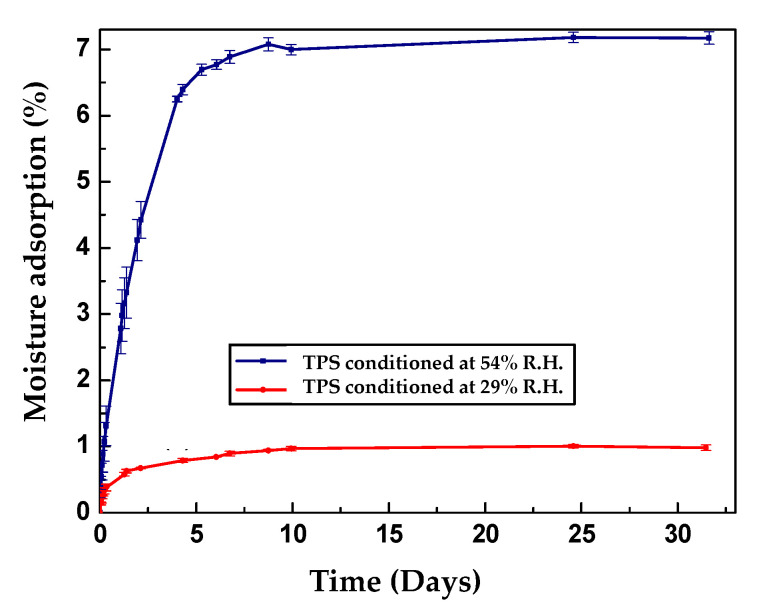
Adsorption isotherms at different storage times for TPS samples conditioned at 29% and 54% relative humidity.

Figure 4 shows the graphs corresponding to the adsorption isotherms for the TPS samples conditioned at 29% and 54% relative humidity and different storage times. In both cases, the slope of the curve that is associated with the rate of adsorption of the material shows that during the first days of conditioning, the increase in mass by the adsorption of moisture occurs very quickly. Subsequently, this rate of adsorption decreases until the material reaches equilibrium moisture adsorption values of approximately 1% and 7% for the samples conditioned at 29% and 54% relative humidities, respectively. It is important to comment that although the adsorption results achieved were very low, the moisture contents in TPSs strongly depend on the nature of the starch used, the content and type of plasticizer, the relative humidity, and the conditioning time, as well type of oven used and the drying conditions before the stabilization of the material, among others. For this reason, in the scientific literature, the reported percentages of adsorption vary over a wide range, as seen in the data recorded in Table 2.

**Figure 5:**
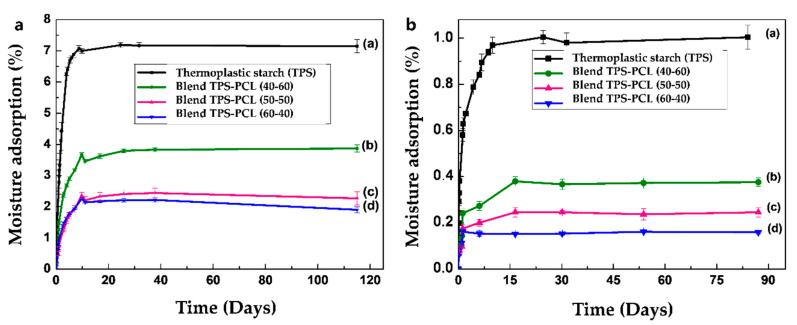
Moisture absorption of TPS, TPS/PCL 60/40 binary blend, TPS/PCL 50/50 binary blend, and TPS/PCL 40/60 binary blend, conditioned at (**a**) 54% and (**b**) 29% relative humidity.

Figure 5a,b show the graphs corresponding to the adsorption isotherms for the samples of the TPS/PCL binary blends at the three proportions after conditioning at 29% and 54% relative humidities, respectively. As expected, in both cases, the adsorption of moisture at equilibrium decreases as a direct function of the amount of incorporated PCL. This has to do with the greater capacity of moisture adsorption exhibited by the TPS since it has hydroxyl groups in its structure that act as proton acceptors and/or donors. These groups increase the potential to interact with the humidity of the environment to a much greater degree than that of PCL, which has carbonyl groups in its structure. On the other hand, the binary blends in all the compositions evaluated show a greater amount of moisture adsorption when they are conditioned to 54%, which is consistent with the highest abundance of water in the environment available to interact with the material, in comparison with samples that are conditioned at 29% relative humidity.

### 4.4. Thermogravimetric Analysis (TGA)

**Figure 6:**
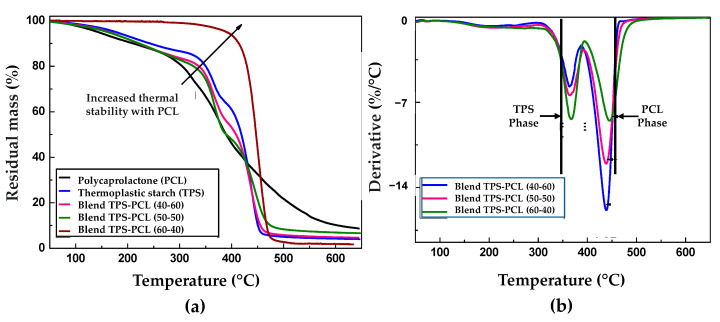
Thermogravimetric (TG) curves for (**a**) neat PCL, neat TPS, and binary blends TPS/PCL. DTG curves for (**b**) binary blends TPS/PCL.

Figure 6 shows the results of the thermogravimetric analysis of the PCL, the TPS, and the binary mixtures TPS/PCL to the three incorporated contents (60/40, 50/50, and 40/60). The thermograms in Figure 6a show the greater thermal stability achieved in the PCL, where the onset of mass loss is above 300 °C. Some research suggests that a two-stage mechanism degrades PCL, a polymer chain cleavage via cis-elimination, followed by unzipping depolymerization from the polymer chain’s hydroxyl end [48]. On the other hand, in TPS, the lowest thermal stability was presented, generating mass decreases from low temperatures, mainly due to the loss of volatile compounds such as water and glycerol. During the thermal degradation process of TPS, three important thermal events occur, which are related to an initial mass loss by water desorption, followed by the dehydration of the hydroxyl groups that are close to the glucose ring, and finally, at high temperatures, the break of the main chain occurs. These regions have been widely reported [28,49]. With the incorporation of polycaprolactone to prepare binary blends, the onset was shifted towards higher temperature values, which increased the percentage of PCL introduced. With the study of the derivative of the thermogravimetric analysis (DTG) of the TPS and the PCL, it was found that the temperature at which the greater mass loss rate was presented for each material was 373 °C and 453 °C (represented with black lines in Figure 6b), respectively. In the case of the DTG curves of the TPS/PCL mixtures (Figure 6b), a first broad and low-intensity peak between 150 °C and 250 °C is observed, associated with the evaporation of the glycerol used as a plasticizer. Later, between 320 °C and 390 °C, a second pronounced peak is related to the principal starch decomposition and decreases its intensity by increasing the binary blend’s PCL content. Finally, between 400 °C and 470 °C, a third peak is present, which shows the PCL decomposition, highlighting an increase in its intensity with the increase of the binary blend’s PCL content. These results are similar to those reported by Correa et al., 2017 [3]. Likewise, the peaks corresponding to the TPS and PCL phases that occur separately in the binary blend only show a slight displacement concerning the signals that occur in the pure TPS and PCL, which highlights the predominantly immiscible nature of the constituent materials, which had previously been established from the analysis of the results obtained using FTIR and SEM characterization. Similarly, in other works [23,50], this immiscible nature of the TPS/PCL mixture has also been reported.

### 4.5. X-ray Diffraction (XRD)

**Figure 7:**
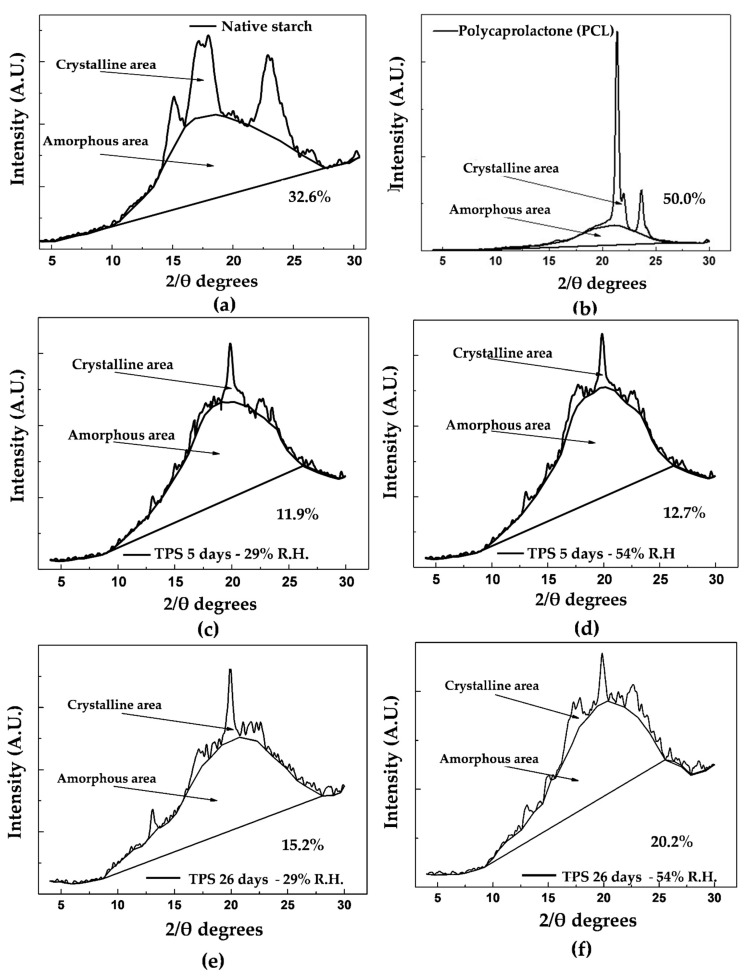
Crystalline and amorphous areas of (**a**) native starch, (**b**) PCL, (**c**) TPS at 5 days of storage and 29% relative humidity, (**d**) TPS at 5 days of storage and 54% relative humidity, (**e**) TPS at 26 days of storage and 29% relative humidity, (**f**) TPS at 26 days of storage and 54% relative humidity.

Figure 7a shows the X-ray diffractogram for native cassava starch (unplasticized). Here, we can see the main 2θ angles that characterize the diffraction planes of crystalline form C, typical of starches from roots as a botanical source [51]. Similarly, Table 3 shows the main signals found for this material, highlighting the peaks at 15.1°, 17.2°, 18.0°, and 22.9° 2θ generated at medium and high intensities. Some of these bands have also been reported by Van Soest et al. [52], who worked with native corn starches that presented type C crystallinity.

**Figure 8:**
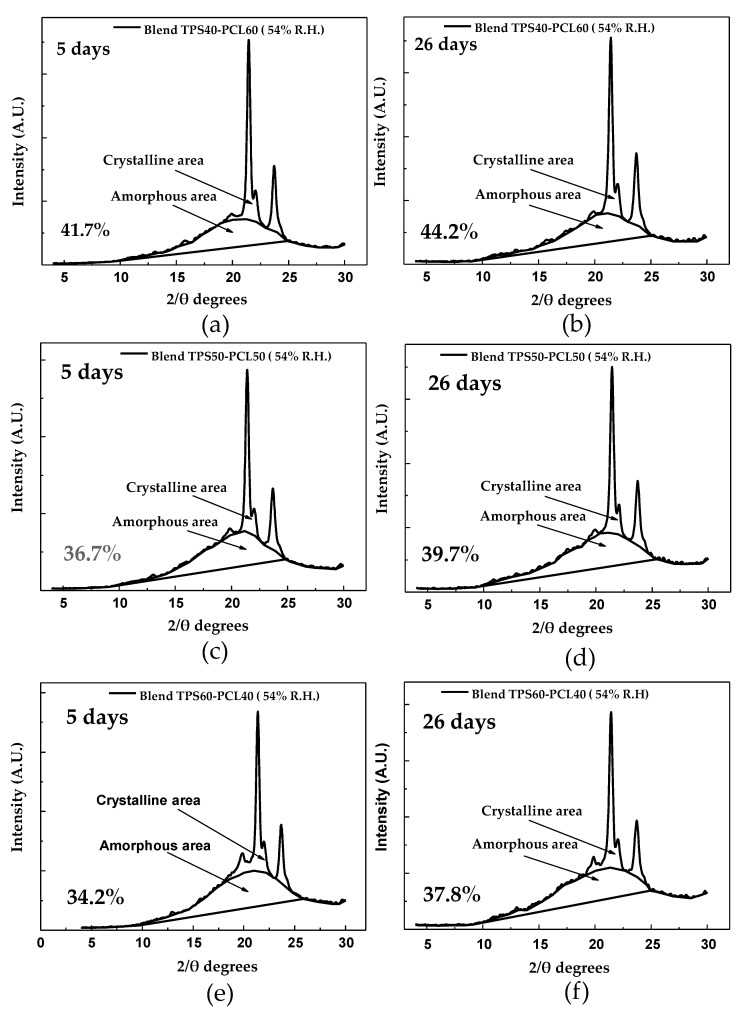
Crystalline and amorphous areas of: (**a**) TPS/PCL 40/60 binary blend conditioned at 5 days; (**b**) TPS/PCL 40/60 binary blend conditioned at 26 days; (**c**) TPS/PCL 50/50 binary blend conditioned at 5 days; (**d**) TPS/PCL 50/50 binary blend conditioned at 26 days; (**e**) TPS/PCL 60/40 binary blend conditioned at 5 days; (**f**) TPS/PCL 60/40 binary blend conditioned at 26 days.

The portions corresponding to the amorphous and crystalline zones, highlighted in Figure 7a, serve as a basis for the estimation of a relative crystallinity of 32.6%, following the method of Nara and Komiya [31]; this percentage is slightly lower than the 38% reported by Atichokudomchai et al. [53] in cassava starch. However, note that this parameter depends on the cassava variety and the amylose and amylopectin contents; therefore, it should not necessarily be the same for materials from different continents. Other crystallinity values reported in the literature correspond to relative crystallinities of 36.1% for yam starch [54] and 30.3% for corn starch [28], both estimated by the method proposed by Nara and Komiya [31]. The TPS diffractogram in Figure 7d indicates that the material acquires a predominantly amorphous structure in the plasticization process, significantly decreasing the intensity of the signals corresponding to the diffraction planes found in the starch before plasticization, as shown in Table 3. The trace peaks at 22.79° and 17.7° 2θ are associated with residual crystallinity in the TPS, mainly due to the starch particles that are not completely destructured during the material’s extrusion. After the starch’s plasticization, a new signal appears at 19.8° 2θ, revealing that a different form of crystallinity is generated in the material. This phenomenon can be attributed to the rearrangement of the polymer chains, mainly amylose, by forming double helices during the extrusion plasticization process. The orientation induced by extrusion leads to a new crystalline arrangement and, therefore, planes that diffract the previously mentioned signal. Similar results were reported by Van Soest et al. [52], who studied the retrogradation of potato starch and found that this new crystalline phase corresponded to the V_H_ type associated with amylose crystals with a simple helical structure. The estimated relative crystallinity for the TPS after storage for 5 days at 54% relative humidity is 12.7%. As the storage time increases, the TPS experiences an increase in crystallinity to a value of 20.2%, as observed in the diffractogram corresponding to the TPS at 26 days of storage (Figure 7f). Here, there are structural changes due to the polymer chains’ rearrangements, causing small increases in the intensities of the signals at 22.7°, 15.1°, and 17.8° 2θ, which in turn indicates an increase in the diffraction planes corresponding to the crystalline form C of native starch. For the samples stored at 29% relative humidity (Figure 7c), the trend is similar to that found for the samples stored at 54%, with the fundamental difference that the intensity of the peaks in the diffractogram taken at 26 days, which are related to the retrogradation process, is lower concerning those obtained in the material stored at 54% relative humidity, as seen in a comparison of Figure 7c,d with Figure 7e,f. In turn, the relative crystallinity reached at 5 days is 11.9%, which increases to 15.2% at 26 days of storage, and these values are lower than those generated in the samples subjected to 54% relative humidity. As previously discussed, based on the mechanical results, this indicates that the humidity of the environment plays an important role in the retrogradation process; relatively high humidity favors this process because it increases the chains’ mobility and the probability that the material can be rearranged over time. Figure 7b shows the X-ray diffractogram corresponding to pure PCL, where three peaks corresponding to angles 21.4°, 22.0° and 23.6° (2θ) are identified, which are similar to those reported by Ortega-Toro et al. [5], who also reported that these signals are associated with diffraction planes (110), (111) and (200) corresponding to an orthorhombic crystal structure. Based on the spectrum found, a relative crystallinity percentage of 50% is estimated using the Nara and Komiya method [31]. This crystallinity value is similar to the 44% reported by Ortega-Toro et al. [5], also by XRD; to the 54% reported by Choi et al. [55]; and to the 46% by Rosa et al. [56], determined by differential scanning calorimetry (DSC). In the binary blends with TPS, a decrease in crystallinity is observed concerning pure PCL. This decrease increases as the PCL content in the blend decreases, going from 41.7% to 34.2% relative crystallinity for the TPS/PCL binary blends of 40/60 and 60/40, respectively (measured at 5 days of storage). In contrast, with increasing storage time from 5 to 26 days, keeping the relative humidity constant at 54%, an increase in crystallinity is observed in all the binary TPS/PCL blends, as revealed by the diffractograms in Figure 8a–f. This increase is 2.5% for the TPS/PCL binary blend (40/60), 3% for the TPS/PCL binary blend (50/50), and 3.6% for the TPS/PCL binary blend (60/40). Under the storage conditions used, PCL does not exhibit significant structural changes, so the variations in crystallinity exhibited by the binary blends are attributed exclusively to TPS’s structural rearrangements, which is supported by the relative crystallinity data. Similarly, no interactions are detected between TPS and PCL by FTIR spectroscopy, which shows that the incorporation of PCL does not appreciably affect TPS’s behavior in the binary blend.

### 4.6. Tensile Strength

**Figure 9:**
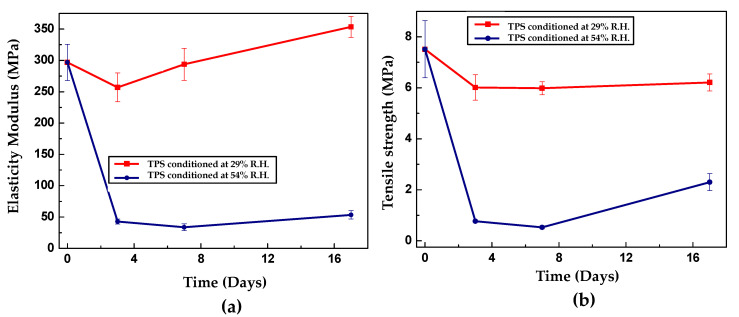
Tensile mechanical properties for TPS as a function of storage time at 25 °C and relative humidity of 54 and 29%. (**a**) Modulus of elasticity and (**b**) maximum strength.

The mechanical behavior evaluated by tensile tests allows the establishment of the dependence of the strength and modulus of elasticity of TPS on the storage time for relative humidities of 29% and 54% at constant temperature (25 °C). Figure 9a,b show the behavior of the maximum strength and modulus of elasticity for the different storage times. Despite its initial decrease from 297 to 43 MPa, Young’s modulus is the parameter that varies the least with the retrogradation phenomenon that prevails at prolonged storage times. As shown in Figure 9b, the modulus tends to increase with time but at a lower rate than the maximum resistance. Note that in the next section, the XRD results establish that the crystallinity of TPS increases from the beginning of storage, mainly due to the recrystallization of the crystalline form C of the native starch. In this sense, an increase in the material’s strength during the entire storage period is expected due to the greater stiffness associated with a more ordered structure. However, the initial decline in the mechanical performance, evidenced in the experimental tests, contradicts this idea. This implies that the explanation for this behavior is necessarily based on the competition of two opposite phenomena: on the one hand, retrogradation tends to stiffen the material, and on the other hand, plasticization by moisture adsorption promotes its softening. Initially, the influence of the latter mechanism prevails, at times shorter than approximately 7 days. Subsequently, when the material approaches its equilibrium adsorption, increases in its mechanical strength begin to occur, indicating that at this level, retrogradation becomes the dominant phenomenon. Van Soest and Knooren [57] found similar mechanical behavior when studying potato TPS at different storage times. The authors attributed the changes in the properties to the formation of helical structures and crystals, which reinforced the TPS by establishing a type of physical cross-linking. In a similar study, Van Soest et al. [58] concluded that the change in the mechanical performance of potato TPS was due to recrystallization to form B of the native starch, while they also found that the crystallinity induced by extrusion remained unchanged. In the TPS samples stored at 29% relative humidity, higher strengths, and moduli than those obtained in the test specimens stored at 54% are found. As expected, this behavior is based on the fact that the plasticizing effect of the water is weaker because its abundance is a direct function of the relative humidity in the desiccators. However, the samples’ tendency as a function of time is similar to that of the samples stored at higher humidity: an initial decrease in the maximum strength and modulus of elasticity under stress in the first days of storage. The fundamental difference is that the magnitude of the change in strength experienced by the specimens adjusted to 29% relative humidity is lower because there is little moisture to influence the plasticization process and reduced mobility, making retrogradation less significant. It is not easy to compare the results obtained for the tensile mechanical properties with the values reported in the scientific literature because of the large variations in the data reported in various studies, as shown in Table 4.

**Figure 10:**
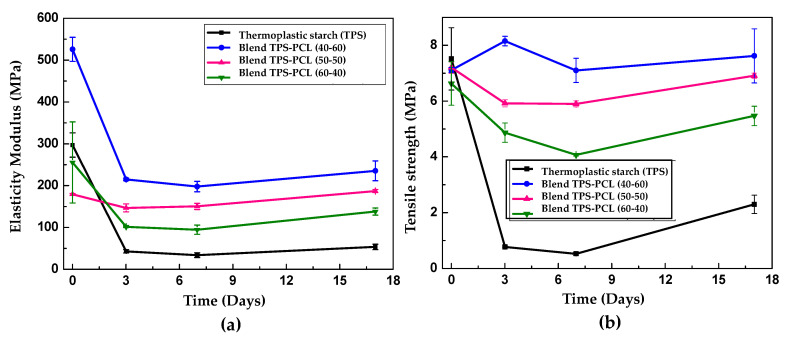
Tensile mechanical properties of TPS/PCL binary blends at 40/60, 50/50, and 60/40 ratios as a function of storage time, at 25 °C, and a relative humidity of 54%. (**a**) Modulus of elasticity and (**b**) maximum strength.

In all cases, the incorporation of PCL generated increases in the modulus of elasticity and tensile strength of the binary blends, and this increase is a direct function of the amount of incorporated PCL. The values obtained in the mechanical characterization are presented in Table 5. For a TPS/PCL blend at a 40/60 ratio, Ishiaku et al. [38] reported a modulus and a maximum tensile strength of 213 and 9 MPa, respectively. These values are similar to those found in the present study, although the researchers used a cross-head displacement speed of 50 mm/min and did not indicate the relative humidity or the test specimens’ storage time. In turn, Averous et al. [23], for a TPS/PCL blend at a 60/40 ratio stored for two weeks at 23 °C and 50% relative humidity, obtained values of 71 and 5.2 MPa for the elastic modulus and maximum strength; these data are similar to those obtained in the present study (138 and 5.5 MPa), reported in Table 4. The elastic modulus obtained in the binary blends presents an initial decrease over the first three days of storage, as shown in Figure 10a.

Subsequently, there are slight increases in this mechanical parameter at longer storage times, following a trend similar to that exhibited by pure TPS. According to the results obtained, it is possible that the phenomena of plasticization by moisture and subsequent stiffening by retrogradation, which occurs in pure TPS, continue to be maintained in the binary blend, being a behavior that occurred independently of the amount of incorporated PCL. This finding may be related to the morphology observed in the SEM images shown in Figure 3a–c, where it is clear that the TPS constitutes a dispersed phase embedded in a PCL matrix, which ultimately implies that the TPS/PCL physicochemical interactions occur only in the periphery of the TPS particles. Therefore, a self-association prevails inside the TPS, and the retrogradation process continues without a significant influence from PCL, so the structural changes in TPS cannot be avoided. Ortega-Toro et al. [4] reported that changes in the tensile mechanical properties of TPS/PCL binary blends with starch contents between 30 and 40% were generated over time, attributing this behavior to TPS retrogradation and the weak interfacial forces that occur with the PCL. In a complementary manner, according to the previous FTIR analysis, no hydrogen bonds are formed between the TPS and PCL, which could contribute to greater compatibility and/or miscibility. The variation in the maximum tensile strength for the different storage times is presented in Figure 10b; here, a trend similar to that shown by the elastic modulus can be observed, and this change is attributed to the same reasons discussed above.

## 5. Conclusions

The mechanical properties and the decrease in TPS moisture adsorption increased with the incorporation of PCL in binary blends, and this increase was a direct function of the amount of PCL added. This behavior indicates that according to the application of interest (biodegradable devices, standing out on drug delivery, tissue engineering, and packing), it is possible to increase the TPS/PCL binary blend’s mechanical properties with other PCL additions. Similarly, the thermogravimetric analysis showed that the binary blend’s thermal stability improved with the incorporation of the PCL, which was the most stable component. It was also shown that the transitions corresponding to the TPS and PCL components were preserved at the peaks of the DTG, thus highlighting the immiscible nature of the TPS/PCL blend. Over time, the changes in the mechanical properties followed a trend similar to that found for pure TPS, that is, the competition of two opposite phenomena. An initial decrease in the mechanical behavior by the plasticization generated by moisture adsorption and an increase in these properties by retrogradation, with the first mechanism predominating at shorter storage times and subsequently reversing for longer times when retrogradation prevailed. This conduct suggested that within the binary blend, the phase corresponding to TPS that was found dispersed in the PCL matrix continued changing structurally in a way similar to that of TPS alone. The study of the crystallinity by XRD confirmed that the incorporation of PCL on the retrogradation of the binary blends was negligible, corroborating that the secondary interactions between the TPS and PCL phases were not significant enough to buffer the structural changes that continued to occur within the TPS. The latter was validated with FTIR, where results showed no relevant interactions between the binary blends’ components.

## Figures and Tables

**Table 1:** Characteristic bands and type of bond for the TPS/PCL binary blend (50/50).

Functional Group/Assignment	Group Frequency, Wavenumber (cm^−1^)	
	Experimental Dates	Balmayor et al. [32]
Hydroxyl O–H stretch of the starch	3331	3362
Methylene CH_2_ asymmetric/symmetric stretch of the polycaprolactone	2945/2866	2944/2864
Carbonyl C=O stretch of the polycaprolactone (ester)	1724	1724
C–O single bond C–O–C stretch of the polycaprolactone	1242	1244
Glycosidic C–O–C stretch of the starch	1043/1029	1048/1021

**Table 2:** Moisture adsorption values reported for thermoplastic starches at different storage conditions.

Materials Starch/Plasticizer	* Plastizicer Content (%)	Conditions for the Adsorption Determination			Moisture Adsorption (%)	Reference
		Drying Temperature (°C)	Drying Time (hours)	Relative Humidity		
Corn/Glycerol	23	105	12	43	9.0	Curvelo et al. [16]
				100	65.0	
Corn/Glycerol	23	105	12	75	37.0	Ma et al. [39]
Corn/Urea-ethanolamine	23	105	24	25	16.0	Huang and Yu [40]
				50	23.0	
Wheat/Glycerol	30	N.R.	N.R.	90	35.0	Liu et al. [41]
Potato/Glycerol	23	105	N.R.	53	13.0	Thunwall et al. [42]
Corn/Glycerol	23	105	12	100	46.0	Ma et al. [43]
Cassava/Glycerol	38	N.R.	N.R.	53	11.2	Teixeira et al. [44]
Corn/Glycerol	23	110	12	53	11.6	Da Róz et al. [45]
				97	60.0	
Corn/Glycerol	** 15	110	36	100	30.7	Zhang et al. [46]
Cassava/Glycerol	30	80	12	54	10.5	Mina et al. [47]
Cassava/Glycerol	30	60	24	54	7.0	Present work
Cassava/Glycerol	30	60	24	29	1.0	Present work

* Percentage calculated with respect to the total mass (plasticizer + starch). ** Percentage calculated with respect to the mass of the hydrolyzed starch. N.R.—not reported.

**Table 3:** Data corresponding to the diffraction peaks of native starch, TPS, and PCL.

Material	Angle (°, 2θ)		Intensity	Type
	Experimental Dates	Reference		
**Native starch**	15.1	N.R. [52]	Very strong	C
	17.2	17.6 [52]	Strong	
	18.0	N.R. [52]	Strong	
	22.9	22.6 [52]	Strong	
	30.2	30.2 [52]	Weak	
	33.5	33.5 [52]	Weak	
**Thermoplastic starch (TPS)**	13.0	13.0 [52]	Medium	V_H_ with Residual crystallinity C
	17.7	N.R. [52]	Weak	
	19.8	19.8 [52]	Very strong	
	22.7	N.R. [52]	Weak	
**Polycaprolactone (PCL)**	21.4	21.6 [5]	Very strong	Planes (110)
	22.0	22.2 [5]	Weak	Planes (111)
	23.6	23.3 [5]	Strong	Planes (200)

N.R.—not reported.

**Table 4:** Tensile mechanical properties of different TPS samples under different storage conditions. Percentage calculated with respect to the total mass (plasticizer + starch).

Material Starch/Plasticizer	Relative Humidity (%)	Crosshead Rate (mm/min)	Tensile Strength (MPa)	Modulus of Elasticity (MPa)	Tensile Strain (%)	Reference
Corn/23% Glycerol	50 (14 days)	10.0	5.5	38.1	7.0	Huang et al. [1]
Corn/23% Glycerol	60 (14 days)	50.0	5.0	125.0	N.R.	Curvelo et al. [16]
Potato/23% Glycerol	70 (14 days)	10.0	5.4	38.0	27.0	Van Soest et al. [58]
Corn/Water	17 (7 days)	50.0	16.7	830.0	3.3	Shogren [59]
Potato/29% Glycerol	57 (2 days)	2.0	5.0	N.R.	4.0	Lourdin et al. [60]
Corn/23% Urea-Ethanolamine	50 (14 days)	10.0	6.4	124.7	116.7	Huang y Yu [40]
Starch/30% Glycerol	N.R.	N.R.	0.4	N.R.	68.9	Ruiz [61]
Corn/23% Urea	33 (7 days)	10.0	12.6	1664.0	5.7	Ma et al. [15]
Corn/23% Ethanolamine	33 (7 days)	10.0	3.1	57.0	61.6	
Corn/23% Urea-Ethanolamine	33 (7 days)	10.0	9.0	236.0	34.4	
Corn/20% Glycerol	50 (7 days)	10	4.8	N.R.	85.0	Yu et al. [62]
Corn/ * 15% Glycerol	100 (N.R.)	10	2.1	N.R.	16.2	Zhang et al. [46]
Corn/23% Glycerol	N.R. (7 days)	10	4.5	80.0	N.R.	Ma et al. [43]
Starch/38% Glycerol	53 (10 days)	50	1.8	16.8	29.8	Teixeira et al. [44]

* Percentage calculated with respect to the mass of the hydrolyzed starch. N.R.—not reported.

**Table 5:** Tensile mechanical properties for TPS, PCL, and TPS/PCL binary blends at 40/60, 50/50, and 60/40 ratios, stored at 54% relative humidity and 25 °C for 17 days.

Materials	Tensile Strength (MPa)	Modulus of Elasticity (MPa)
Polycaprolactone (PCL)	23.9	383.0
Binary blend TPS/PCL (40/60)	7.6 ± 1.0	235.3 ± 23.8
Binary blend TPS/PCL (50/50)	6.9 ± 0.1	187.0 ± 3.6
Binary blend TPS/PCL (60/40)	5.5 ± 0.4	138.0 ± 8.5
Thermoplastic starch (TPS)	2.3 ± 0.3	53.5 ± 6.6
